# Survey among Italian experts on existing vaccines’ role in limiting antibiotic resistance

**DOI:** 10.1080/21645515.2021.1969853

**Published:** 2021-09-30

**Authors:** Federico Marchetti, Rosa Prato, Pierluigi Viale

**Affiliations:** a GSK, Verona, Italy; b Department of Medical and Surgical Sciences, University of Foggia, Foggia, Italy; c Department of Hygiene, Policlinico Riuniti University Hospital of Foggia, Foggia, Italy; d IRCCS Policlinico Sant’Orsola, Infectious Disease Unit, Department of Medical and Surgical Sciences, University of Bologna, Bologna, Italy

**Keywords:** Antibiotic resistance, Italy, measles, MenACWY, MenB, meningococcal, vaccination, vaccines, varicella, pertussis

## Abstract

Antimicrobial resistance (AMR) is a major public health problem threatening to reverse the progress made against infectious diseases. The rapid increase of AMR exposes Italian hospitals at increased risk of untreatable infections. Vaccinations can potentially limit AMR by reducing the number of infected cases in need of antibiotics. We conducted a survey among Italian vaccine experts to record their opinion regarding the role of vaccinations against antibiotic resistance (ABR). Among 80 invited experts, 51 answered all questions. Most respondents were experts in hygiene and preventive medicine (56.9%) and aged >50 years (72.6%). ABR was a priority concern in the daily professional activity of 82.4% of respondents. Overall, 47.1% of respondents believed that all vaccinations included in the vaccination calendar played a role against ABR: 92.2% for pertussis vaccination followed by 88.2%, 74.5%, and 70.6% for meningococcus, measles, and varicella vaccinations, respectively. Almost all respondents agreed that the role of vaccinations against ABR should be clearly expressed in the national vaccination guidelines (96.1%) and Scientific Societies should take an explicit position on the issue (92.2%). These results show that Italian experts have recognized the vaccinations’ potential role in limiting ABR and guidelines from the appropriate scientific and governmental authorities are needed.

## Graphical abstract

**Figure:**
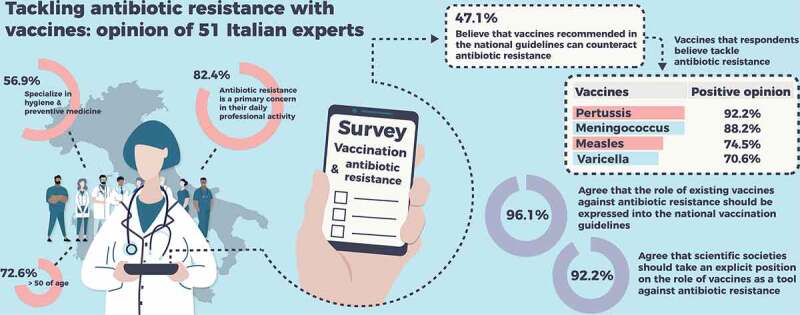

## Introduction

Antimicrobial resistance (AMR) is a major public health problem that threatens to undermine the effectiveness and sustainability of modern medicine.^1,2^ Globally the increasing AMR raises the risk of a near future dominated by an ominous shortage of effective antibiotics against several bacteria.^3,4^

AMR develops in response to the “selective pressure” imposed to pathogens by the antimicrobials, for instance, antibiotics, antivirals, antifungals, and antiparasitics.^5,6^ Following a Darwinian evolutionary process, in the presence of antimicrobials, surveilled bacteria, viruses, fungi, and parasites that can evolve over time so as to achieve survival and reproduction into resistant progeny can progressively (and sometimes rapidly) replace the previous nonresistant community.^5,6^ The involved molecular mechanisms are variable and complex, including enzymatic degradation of antibiotics, increased efflux pump activity, alteration in metabolic pathways, membrane modifications decreasing permeability by antibiotics, and other genetic modifications.^5^ Pathogens displaying such AMR mechanisms have been reported by healthcare systems and community globally,^7^ and continuously evolve, often resulting in simultaneous resistance against several classes of antibiotics.^5^

The AMR problem has been observed and recognized by scientists since the beginning of the antibiotic era.^8,9^ However, it is only during the last 25 years of systematic misuse and overuse of antibiotics that AMR had become a life-threatening issue.^7,9^ According to the latest estimates of the Organization for Economic Co-operation and Development, 2.4 million deaths due to AMR will occur from 2015 to 2050.^10^ This may be an underestimate: in the European Union (EU) and European Economic Area (EEA) alone, 670,000 infections leading to 33,000 deaths occur each year.^11,12^ The associated societal cost is estimated at €1.5 billion.^3^ Almost one out of five of these infections is due to AMR.^12^ AMR to last-line antibiotics accounts for 40.0% of the overall AMR-induced healthcare burden in the EU/EEA countries.^12^ This is a serious problem, considering that it is extremely difficult if not impossible to treat infected patients resistant to last-line antibiotics.^12^ The frequency and patterns of AMR vary across Europe.^12^ In Italy, AMR has taken alarming dimensions with the country reporting one of the highest incidences in Europe.^13^ According to the 2019 European Center for Disease Prevention and Control (ECDC) surveillance data, Italy is among the countries with highest percentage of resistant isolates for under surveillance bacterial species such as *E. coli, K. pneumoniae, S. aureus* etc.^11^ According to the ECDC data, on intensive care units (ICUs) in Italy there are records for: (a) high incidence of intubation-associated pneumonia and central venous catheter-associated bloodstream infections, (b) longer length of stay, and (c) longer durations of intubations and catheter use.^13^ Gram-negative bacteria resistant to multiple antibiotics are prevalent among ICU infections across Italy.^13^

In 2015, the World Health Organization (WHO) introduced the “One Health” strategy under which stakeholders from human and veterinary health, agriculture, environmental research, and economics, joined efforts to fight AMR.^14^ As a result, an Action Plan was framed to increase awareness, prevent infections, optimize antibiotics use, and, finally, to promote sustainable investments in new medical interventions.^14^ WHO called on supranational and individual country institutions to put in place measures against AMR^15^ and a Global Plan to fight AMR.^14^ The EU, in turn, issued directives to member states to confront AMR,^16^ emphasizing the prudent use of antibiotics.^17^ In Italy, the Ministry of Health has, for some years, prepared and coordinated a series of measures to limit AMR.^18^ These measures, framed under the “One Health” strategy, have been included in the National Action Plan to Combat Antimicrobial Resistance (Piano Nazionale di Contrasto dell’Antimicrobico-Resistenza [PNCAR] 2017–2020).^19^ Within the PNCAR, a collaboration was mandated between central, regional, and local multi-sector institutions to: (a) improve levels of awareness and information/education in health professionals, citizens, and stakeholders; (b) survey AMR incidence; (c) improve infection control and prevention; (d) optimize the use of antimicrobials; and (e) support AMR-related research.^18,19^ The PNCAR incorporates vaccinations in the list of topics related to AMR but only acknowledges their role in reducing acute viral (e.g., influenza or measles) and bacterial (e.g., pneumococcal) infections, and therefore on care-related circulation of antibiotic-resistant strains.^19^ PNCAR does not provide explicit guidance or objectives for healthcare providers (HCPs) and other stakeholders on vaccinations in relation to AMR, for instance,i.e. specific populations where the risk of selection of resistant bacteria is higher and hence, high vaccination coverage should be reached.^19^

Toward addressing the need for prevention, the “One Health” strategy encourages vaccinations with existing vaccines as well as the development of new or improved vaccines for infections difficult to treat due to AMR.^14^ In a recent publication, WHO further details the contribution of vaccines as a weapon against AMR by preventing infections and reducing the need for antibiotics.^2^ Vaccination can decrease AMR prevalence by various pathways. Primarily, vaccination reduces the number of infected cases in need of antibiotic treatment.^20^ Misuse of antibiotics,^21^ and empirical administration of broad-spectrum antibiotics to treat clinical syndromes such as pneumonia^22^ should also be reduced through effective vaccination programs. Furthermore, by preventing infections, pathogens have fewer chances to proliferate and thus fewer opportunities to develop resistant strains.^21^ Moreover, through herd immunity, the contacts of vaccinated individuals are less likely to acquire infections, which may require antibiotic therapy.^22^ Vaccinations also protect the microbiome from disruption induced by broad-spectrum antibiotics and therefore the development of resistant “bystander” bacterial species by the acquisition of resistance genes from other organisms in the microbiome.^21,22^ Finally, by reducing hospitals overcrowding during epidemic periods (like influenza season), vaccines could help lower intra-hospital bacteria circulation.^23^

Published evidence supports the role of vaccinations in limiting AMR, particularly for influenza^24^ and pneumococcal^25^ vaccines. Currently only clinical studies of influenza and pneumococcal vaccines have generated sufficient data for a full analysis on the use of antimicrobials following vaccination.^26^ These data indicate that influenza and pneumococcal vaccinations significantly reduced antimicrobial use in the groups of vaccinees and their household contacts.^26^ Additional high-quality research data concerning all other vaccinations are needed and should be collected in future vaccine trials.^27^ Nevertheless, recording experts’ opinions on the role of vaccines other than those for influenza and pneumococcal in reducing AMR contribute to the general knowledge on the topic. By definition, clinical expertise is an integral part of evidence-based medicine (EBM), together with best research evidence and patients’ values.^28^ To provide a first EBM assessment step, a survey to document the value attributed by experts in the field of vaccination to four vaccinations included in the National Preventive Vaccination Plan (Piano Nazionale Prevenzione Vaccinale [PNPV], in which the National Vaccination Calendar is incorporated)^29^ in combating antibiotic resistance (ABR) was carried out.

## Methods

### Study design

A survey questionnaire was deployed online selected *vaccine experts* across the country, from June 26, 2020 to July 31, 2020.

The survey was focused on ABR, which was considered more meaningful for the four selected vaccinations: meningococcal (serogroup B meningococcal [MenB] and meningococcal conjugate, quadrivalent A, C, W, and Y [MenACWY]), pertussis, measles, and varicella.

A personal e-mail invitation to participate in the survey was sent and answers were collected anonymously. The questionnaire consisted of 16 questions (Supplementary material): 12 multiple-choice questions and four qualitative response questions on a Likert-type scale from 1 (very little) to 10 (very high).

### Participants

Participants were *vaccine experts*, for instance, professionals with a prominent role in academia or the healthcare system with activities that were linked to the field of vaccinology or public health: for example, authors of relevant peer-reviewed publications, speakers in vaccinology or public health topics at congresses, holding relevant institutional positions, or recognized within the Scientific Societies they belonged to.

A total of 80 external experts in hygiene and preventive medicine, pediatrics, infectious diseases, and similar were identified and personally invited to take part in the survey.

### Statistical methods

A descriptive analysis of the sample was conducted. Categorical responses were reported as frequencies and percentages. Bar graphs and tables were used to summarize statistics. For the answers to the Likert scale, the median and interquartile range (IQR) were calculated and represented graphically with box plots. The statistical package Stata/IC Version 15.1 (StataCorp LLC, College Station, TX, USA) was used for data analysis.

## Results

Overall, 80 experts were invited, 60 experts connected to the survey and 51 experts answered all questions. Therefore, the survey participation rate was 63.8% (51/80), and the dropout rate was 15.0% (9/60). Most respondents were >50 years old and were experts in hygiene and preventive medicine (Table 1).

**Table 1.** Survey respondents’ characteristics

Characteristics	N = 51 n (%)
Age group, years	
<30	0 (0.0)
30–40	5 (9.8)
41–50	9 (17.6)
>50	37 (72.6)
Geographic region	
North	26 (51.0)
Center	10 (19.6)
South and islands	15 (29.4)
Field of Professional Expertise	
Hygiene and Preventive Medicine	29 (56.9)
Hospital Pediatricians	6 (11.8)
Family Pediatricians	9 (17.6)
Infectious Diseases	2 (3.9)
Other	5 (9.8)

ABR was a priority concern in the daily professional activity of 82.4% of respondents (Supplementary Figure 1A). Stratifying these results by specialty, ABR was a priority concern for all (100.0%) hospital pediatricians and infectious diseases experts, 79.3% of hygiene and preventive medicine physicians, and 77.8% of family pediatricians.

For 92.2% of respondents, the PNPV was a topic often or very often discussed with colleagues, patients, and families (Supplementary Figure 1B).

**Figure 1.**
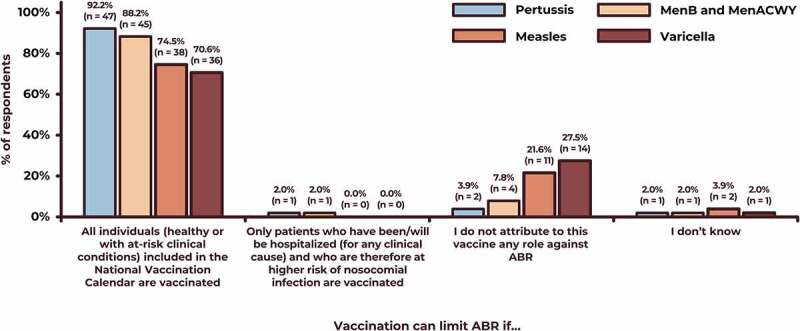
Respondents’ opinion on the role of four vaccines against ABR.

Figure 1 summarizes the respondent’s evaluation of the four vaccinations’ specific role against ABR. Pertussis vaccination of all individuals (healthy or with at risk clinical conditions) could counteract AMR according to 92.2% of respondents; corresponding percentages for the vaccinations with meningococcal (MenB and meningoccocus (MenACWY), measles, and varicella vaccines were 88.2%, 74.5%, and 70.6%, respectively (Figure 1). All (100.0%) hospital pediatricians believed that measles and varicella vaccination could counteract ABR; for the other vaccine types, the same opinion was shared by 50.0%-77.8% and 50.0%-69.0% of all other experts.

ABR antibiotic resistance; MenB, serogroup B meningococcal; MenACWY, meningococcal conjugate, quadrivalent A, C, W, and Y.

**Figure 2.**
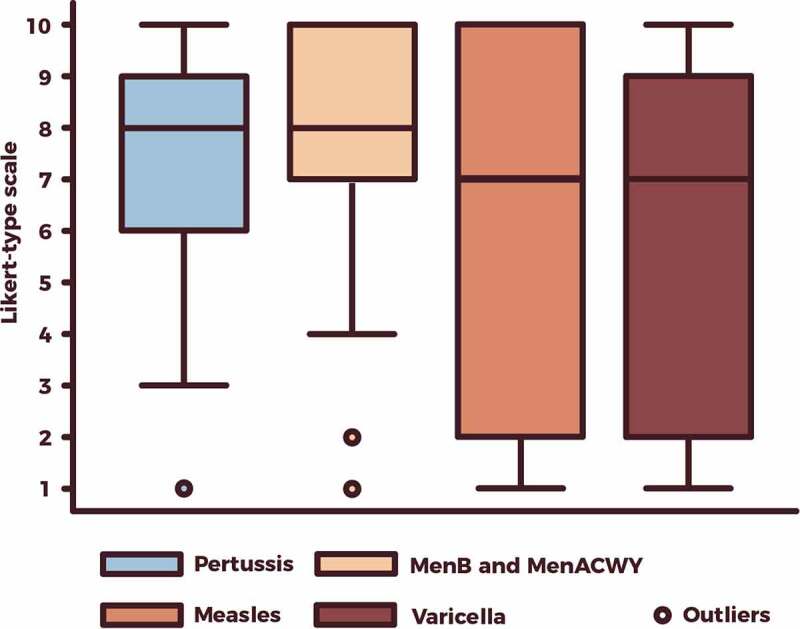
Respondents’ opinion on the role of four vaccines (meningococcal [MenB and MenACWY], pertussis, measles, and varicella) against ABR on a Likert-type scale from 1 (minimum) to 10 (maximum).

ABR, antibiotic resistance; MenB, serogroup B meningococcal; MenACWY, meningococcal conjugate, quadrivalent A, C, W, and Y.

Figure 2 illustrates the Likert-type scale scores corresponding to each vaccination’s role against ABR. On the Likert-type scale the median scores for the role of vaccinations included in the PNPV were 8 (IQR, 6–9) for pertussis vaccination, 8 (IQR, 7–10) for meningococcal (MenB and MenACWY) vaccination, 7 (IQR 2–10) for measles vaccination, and 7 (IQR, 2–9) for varicella vaccination. There was awider dispersion of votes/opinions for viral vaccinations as compared to bacterial ones.

Overall, 47.1% of respondents believed that all vaccinations included in the PNPV could play a role for ABR containment; 33.3% of respondents recognized that role for influenza, pneumococcus, meningococcus, pertussis, measles, and varicella vaccinations, while 17.7% of respondents only recognized influenza and pneumococcal vaccinations (Supplementary Figure 2A).

A total of 96.1% of respondents believed that the PNPV and the PNCAR should clearly express the role of existing vaccinations in counteracting ABR (Supplementary Figure 2B).

Finally, 92.2% of respondents believed that Scientific Societies should officially express themselves on the topic (Supplementary Figure 2C).

## Discussion

The present survey respondents acknowledged the overall relevance of vaccinations included in the National Vaccination Calendar in confronting ABR. Past surveys have accessed physicians’ perceptions about AMR.^30–33^ However, to our knowledge, this is the first survey exploring experts’ opinions about the role of existing vaccines in reducing ABR, from a single country with a relevant (i.e., n = 51) number of respondents. A similar approach, involving 18 international experts, was chosen by Gavi, the Vaccine Alliance (formerly Global Alliance for Vaccines and Immunization) and the WHO Initiative for Vaccine Research to inform their 2018 priorities for vaccine funding.^34^ The Gavi survey experts assigned the highest value regarding AMR to the pneumococcal, typhoid, and malaria vaccines followed by the rotavirus, respiratory syncytial virus, influenza, measles, meningitis, and Haemophilus influenzae type b-containing pentavalent vaccines.^34^ In the present survey, the respondents’ familiarity with vaccination was medium to high, consistent with the invitation criteria. Overall respondents’ engagement with the ABR topic was 82.4%, which is high, but still leaves room for improvement. Similar trends of suboptimal personal engagement on ABR by physicians was shown in a European survey conducted by ECDC from January to March 2019.^30,31^ In that survey, almost all (90.0%) of “prescribers” stated they kept in mind AMR when treating patients, but only 77.0% of prescribers were confident in making antibiotic prescribing decisions and only 69.0% of prescribers had confidence in the available antibiotic guidelines.^30,31^ In the same ECDC survey, 2,167 participants were healthcare professionals from Italy, 890 of which were physicians.^30,31^ Among the Italian respondents, 30.4% of the hospital prescribers and 35.9% of the community prescribers provided at least one antibiotic prescription per week that they would prefer not to administer.^30,31^ In 2017, the ECDC performed a country assessment visit to Italy to discuss AMR-related issues and concluded that high AMR levels were likely accepted as unavoidable by the stakeholders throughout the healthcare system.^13^ Following this visit, ECDC recommended PNCAR should integrate measurable outcomes, shorter deadlines, central supervision, and intersectoral coordinating strategies against AMR.^13^ Aligned to these ECDC recommendations, almost all respondents in the present survey called for institutional and scientific society intervention on the AMR issue. However, currently, the PNCAR^19^ only briefly mentions vaccinations, and the PNPV^29^ does not mention AMR.

Taking into account the opinions of the experts expressed in the present survey, specific guidance on the role of existing vaccines in tackling ABR should be integrated in both the next PNCAR and PNPV update. Similarly, Scientific Societies interested in the AMR problem should take an official position on the issue and produce ad hoc guidance documents.

The respondents of the present survey attributed greater importance to the bacterial diseases’ vaccines (pertussis and meningococcal [MenB and MenACWY]) than to the viral diseases’ vaccines (measles and varicella) as tools against ABR. Such prioritization is not supported by evidence neither by the WHO Action Plan that involves all existing vaccines in the fight against AMR^14^ and would deserve a deeper investigation.

Even though clinical evidence on the impact on ABR of the four vaccinations taken into account in the survey (meningococcal [MenB and MenACWY], pertussis, measles, and varicella vaccinations) is missing, some considerations on their role can be made.

The antibiotic treatment regimens recommended at European level for the treatment of bacterial meningitis are based on high doses of third-generation cephalosporins associated with aminoglycosides or vancomycin for 7–14 days.^35^ These regimens are applied immediately in the presence of clinical suspicion pending laboratory confirmation of the diagnosis.^35^ Fortunately, the number of confirmed diagnoses of meningitis in Italy is low,^36^ but prescriptions take place for all suspected cases and, in Italy, resistance to third-generation cephalosporins in the bacterial species under surveillance is among the highest in Europe.^37^

A disease that is certainly more widespread than bacterial meningitis is measles.^38^ In 2017, an unusual peak of 4,991 cases of measles was recorded in Italy, resulting in complications in 35.8% of cases. Considering only cases of over-infections, 378 cases of pneumonia and 225 cases of otitis occurred, which certainly required antibiotic therapy.^39^ Similar reasoning can be made for chickenpox, a widespread disease for which however, we lack robust surveillance data in Italy. Nevertheless, because chickenpox vaccination in children was introduced in Italy at regional level in 2003^40^ and at national level in 2017,^19^ it is reasonable to assume that today most cases of chickenpox in Italy involve older children or younger adults; therefore there are moderate- to high risk cases of chickenpox with increased likelihood of infectious complications requiring antibiotic therapy. An analysis in hospital admissions of chickenpox cases in England showed that 38.1% developed complications, most frequently bacterial skin infection (11.2%) and pneumonia (4.8%).^41^ The highest percentage of encephalitis, meningitis, and pneumonia were observed in adults and older children.^41^

Pertussis vaccination received the highest score by the present survey respondents. For children and adolescents, protection can be postulated based on PNPV recommendations.^29^ In adults without chronic illnesses, pertussis usually heals without consequences, although unfortunately it is very rarely (if ever) correctly diagnosed.^42^ However, the disease is debilitating for weeks (sometimes months) and complications may occur, especially with advancing age, including pneumonia, sinusitis, and urinary incontinence.^43^ Moreover, patients with chronic respiratory disease have a higher risk of contracting pertussis, which also contributes to the negative evolution of the chronic disease itself.^43^ The latest (2019) ECDC surveillance data released in 2020 reports that 47% of notified cases were detected in subjects older than 30 years, leading to an overall incidence rate for pertussis of around 5–7 per 100,000 among European patients aged ≥30 years, an age group comprising in Italy 42 million people out of the total general population.^44–46^ The epidemiologic picture becomes bleaker if we consider an underestimation factor of pertussis diagnoses equal to 58–93 times measured in the United States of America in individuals aged <50 years (data not available for Italy).^47^ Based on the clinical presentation of pertussis, it can be assumed that most cases receive at least one antibiotic prescription or more if symptoms persist.

In the primary care setting of Italy, based on a 2018 report, a very marked seasonal trend was reported in antibacterial consumption between the winter and summer months, ranging from a minimum of 11.4 defined daily dose (DDD)/1,000 in August to a maximum of 24.5 DDD/1,000 in January. The more frequent use of antibiotics in the winter months correlates with the peaks of influenza syndromes observed in the different years.^48^ These data support the concept that, if there was vaccination coverage for influenza, pneumococcal disease and diphtheria-tetanus-pertussis, booster doses in adults would be increased. This could lead to a consistent reduction in respiratory infectious diseases, especially in winter, with a consequent reduction in antibiotic prescriptions.

In the “One Health” perspective, all HCPs and other stakeholders should strive to recommend vaccinations not only to increase the overall level of health in the population but also to effectively counteract AMR that otherwise is likely to negatively impact human lives in the coming years. Given the rapid and countrywide increase of AMR, Italian hospitals are facing an increased risk of difficulty-to-treat infections following major medical interventions.^13^ This requires an increased sense of urgency at all levels, including establishing meticulous definitions of each stakeholder’s responsibility.^13^ In that respect, WHO has recently established an operational structure dedicated to assessing the assumed function of vaccines as a tool to combat AMR, both in terms of access to already available vaccines and in terms of research and development for new vaccines specifically designed against major multi-resistant pathogens.^49^

Lastly, the pandemic of coronavirus disease 2019 (COVID-19) added one more aspect to the AMR concerns.^50–53^ Risk for bacterial and fungal infections has been detected in overcrowded ICUs admitting critically-ill COVID-19 patients mechanically ventilated, and where secondary infections were found in 50.0% of deceased COVID-19 patients.^36,50^ In addition, concerns were expressed that enormous work pressure on HCPs might relax adherence to hospitals’ antimicrobial stewardship policies.^54^ On the other hand, the disruption of routine medical care could potentially lead to an overall reduction of antibiotics administration.^51,55^ Apparently, there are several reasons for which antibiotic prescribing and AMR may change under the COVID-19 pandemic. However, the question whether AMR will worsen or improve as a consequence of the current COVID-19 pandemic remains to be answered.^56^

### Limitations

The present survey captured the individual experts’ opinion at the time they completed the survey; having been completed at another time or after a scientific forum on AMR, responses might have been different. The present responses represent the perspective of experts dealing mainly with vaccinations, as previously described and confirmed in the survey results. Although the survey was distributed throughout Italy, the majority of the respondents were residing in northern Italy.

## Conclusion

Italian vaccine experts, in the present survey, were generally concerned over AMR. They consider vaccinations as an important tool to confront the problem and would like to receive additional institutional guidance on combating AMR.

**Figure 3.**
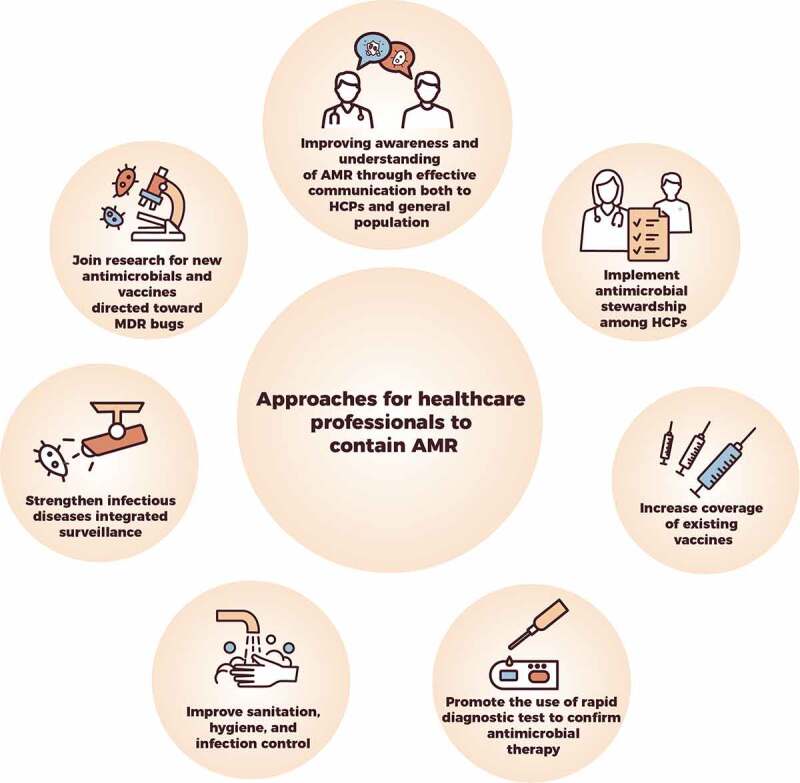
Approaches HCPs can take to tackle AMR.

HCPs, Healthcare professionals; AMR, antimicrobial resistance; MDR, multi-drug resistant.

The present survey was aligned to the WHO Action Framework, which has, among others, the objective to improve “awareness and understanding of the role of vaccines in limiting AMR” by sharing knowledge of vaccine impact on AMR.^2^ Moreover, the high level of participation, uncommon for surveys, reflects a high interest in the topic by the experts on the field. The experts’ opinions expressed in the present survey as derived from their informed daily clinical practice may already constitute preliminary evidence that vaccines contribution has been acknowledged in practice by the specialists in Italy. Vaccinations should be routinely encompassed among actions that HCPs may undertake to tackle AMR (Figure 3). Following this outcome, appropriately designed clinical trials should be conducted to strengthen the currently limited evidence. Moreover, appropriate guidance documents should be developed and communicated to all HCPs in Italy.

## Supplementary Material

Supplemental Material

Supplemental Material

## Figures and Tables

**Table:** 

ABR	antibiotic resistance
AMR	antimicrobial resistance
COVID-19	coronavirus disease 2019
DDD	defined daily dose
EBM	evidence-based medicine
ECDC	European Centre for Disease Prevention and Control
EEA	European Economic Area
EU	European Union
Gavi	the Vaccine Alliance (formerly Global Alliance for Vaccines and Immunization)
HCP	healthcare provider
ICU	intensive care unit
IQR	interquartile range
MenACWY	meningococcal conjugate, quadrivalent A, C, W, and Y
MenB	serogroup B meningococcal
PNCAR	Piano Nazionale di Contrasto dell’Antimicrobico-Resistenza [National Action Plan to Combat Antimicrobial Resistance]
PNPV	Piano Nazionale Prevenzione Vaccinale [National Preventive Vaccination Plan]
WHO	World Health Organization

## References

[1] World Health Organization National Action Plans and Monitoring and Evaluation, Surveillance, Prevention and Control. Global action plan on antimicrobial resistance. [accessed 2020 Feb 19]. https://www.who.int/publications/i/item/global-action-plan-on-antimicrobial-resistance.

[2] Vekemans J, Hasso-Agopsowicz M, Kang G, Hausdorff WP, Fiore A, Tayler E, Klemm EJ, Laxminarayan R, Srikantiah P, Friede M, et al. Leveraging vaccines to reduce antibiotic use and prevent antimicrobial resistance: a WHO action framework. Clin Infect Dis. 2021:ciab062. doi:10.1093/cid/ciab1062. PMC8366823 33493317

[3] European Centre for Disease Prevention and Control (ECDC)/European Medical Agency (EMEA) joint technical report: the bacterial challenge: time to react; 2009 [accessed 2021 Feb 19]. https://www.ecdc.europa.eu/sites/default/files/media/en/publications/Publications/0909_TER_The_Bacterial_Challenge_Time_to_React.pdf.

[4] National Center for Environmental Health; National Center for Health Statistics; National Center for Infectious Diseases, CDC. Achievements in public health, 1900-1999: control of infectious diseases. MMWR Week. 1999;48(29):621–29.

[5] Ferri M, Ranucci E, Romagnoli P, Giaccone V. Antimicrobial resistance: a global emerging threat to public health systems. Crit Rev Food Sci Nutr. 2017;57(13):2857–76. doi:10.1080/10408398.2015.1077192. 26464037

[6] World Health Organization (WHO). Antimicrobial resistance. [accessed 2020 Feb 19]. https://www.who.int/health-topics/antimicrobial-resistance.

[7] Davies J, Davies D. Origins and evolution of antibiotic resistance. Microbiol Mol Biol Rev. 2010;74(3):417–33. doi:10.1128/MMBR.00016-10. 20805405 PMC2937522

[8] Fair RJ, Tor Y. Antibiotics and bacterial resistance in the 21st century. Perspect Medicin Chem. 2014;6:25–64. doi:10.4137/PMC.S14459. 25232278 PMC4159373

[9] Dhingra S, Rahman NAA, Peile E, Rahman M, Sartelli M, Hassali MA, Islam T, Islam S, Haque M. Microbial resistance movements: an overview of global public health threats posed by antimicrobial resistance, and how best to counter. Front Public Health. 2020;8:535668. 33251170 10.3389/fpubh.2020.535668 PMC7672122

[10] Organisation for Economic Co-operation and Development (OECD) Health Policy Studies. Stemming the superbug tide - just a few dollars more; 2018 [accessed 2020 Feb 19]. https://www.oecd.org/health/stemming-the-superbug-tide-9789264307599-en.htm.

[11] European Centre for Disease Prevention and Control (ECDC). Antimicrobial resistance in the EU/EEA (EARS-Net) annual epidemiological report for 2019; 2019 [accessed 2020 Feb 17]. https://www.ecdc.europa.eu/sites/default/files/documents/surveillance-antimicrobial-resistance-Europe-2019.pdf.

[12] Organisation for Economic Co-operation and Development (OECD) and European Centre for Disease Prevention and Control (ECDC). Antimicrobial resistance tackling the burden in the European Union; 2019 [accessed 2020 Feb 19]. https://www.oecd.org/health/health-systems/AMR-Tackling-the-Burden-in-the-EU-OECD-ECDC-Briefing-Note-2019.pdf.

[13] European Centre for Disease Prevention and Control (ECDC). Mission report: ECDC country visit to Italy to discuss antimicrobial resistance issues; 2017 Jan 9-13 [accessed 2020 Feb 19]. https://www.ecdc.europa.eu/sites/default/files/documents/AMR-country-visit-Italy.pdf

[14] World Health Organization (WHO). Global action plan on antimicrobial resistance; 2015 [accessed 2021 Feb 19]. https://www.who.int/antimicrobial-resistance/publications/global-action-plan/en/. 10.7196/samj.9644 26242647

[15] World Health Organization (WHO). Antimicrobial resistance. The Seventy-second World Health Assembly; 2019 May 28 [accessed 2020 Feb 19. https://apps.who.int/gb/ebwha/pdf_files/WHA72/A72_R5-en.pdf.

[16] European Comission. EU action on antimicrobial resistance. [accessed 2021 Feb 26]. https://ec.europa.eu/health/antimicrobial-resistance/eu-action-on-antimicrobial-resistance_en.

[17] Commission notice — EU guidelines for the prudent use of antimicrobials in human health. C/2017/4326. [accessed 2021 Feb 26]. https://eur-lex.europa.eu/legal-content/EN/TXT/?uri=CELEX%3A52017XC0701%2801%29&qid=1614368348994.

[18] Ministero della Salute. antibiotico-resistenza [accessed 2021 Feb 26]. http://www.salute.gov.it/portale/antibioticoresistenza/homeAntibioticoResistenza.jsp.

[19] Piano Nazionale di Contrasto dell’Antimicrobico-Resistenza (PNCAR) 2017-2020. [accessed 2020 Feb 19]. http://www.salute.gov.it/imgs/C_17_pubblicazioni_2660_allegato.pdf.

[20] Micoli F, Bagnoli F, Rappuoli R, Serruto D. The role of vaccines in combatting antimicrobial resistance. Nat Rev Microbiol. 2021;19:287–302. doi:10.1038/s41579-020-00506-3. 33542518 PMC7861009

[21] Buchy P, Ascioglu S, Buisson Y, Datta S, Nissen M, Tambyah PA, Vong S. Impact of vaccines on antimicrobial resistance. Int J Infect Dis. 2020;90:188–96. doi:10.1016/j.ijid.2019.10.005. 31622674

[22] Lipsitch M, Siber GR. How can vaccines contribute to solving the antimicrobial resistance problem? mBio. 2016;7:3. doi:10.1128/mBio.00428-16. PMC4959668 27273824

[23] Bonanni P, Picazo JJ, Remy V. The intangible benefits of vaccination - what is the true economic value of vaccination? J Mark Access Health Policy. 2015;3:26964. 10.3402/jmahp.v3.26964 PMC4802696 27123182

[24] Rodgers LR, Streeter AJ, Lin N, Hamilton W, Henley WE. Impact of influenza vaccination on amoxicillin prescriptions in older adults: a retrospective cohort study using primary care data. PLoS One. 2021;16(1):e0246156. doi:10.1371/journal.pone.0246156. 33513169 PMC7846013

[25] Klugman KP, Black S. Impact of existing vaccines in reducing antibiotic resistance: primary and secondary effects. Proc Natl Acad Sci U S A. 2018;115(51):12896–901. doi:10.1073/pnas.1721095115. 30559195 PMC6304973

[26] Doherty TM, Hausdorff WP, Kristinsson KG. Effect of vaccination on the use of antimicrobial agents: a systematic literature review. Ann Med. 2020;52(6):283–99. doi:10.1080/07853890.2020.1782460. 32597236 PMC7880080

[27] Buckley BS, Henschke N, Bergman H, Skidmore B, Klemm EJ, Villanueva G, Garritty C, Paul M. Impact of vaccination on antibiotic usage: a systematic review and meta-analysis. Clin Microbiol Infect. 2019;25(10):1213–25. doi:10.1016/j.cmi.2019.06.030. 31284031

[28] Sackett DL, Straus SE, Richardson WS, Rosenberg W, Haynes RB. Evidence-based medicine: how to practice and teach EBM. 2nd ed. Churchill Livingstone; 2000. p. 155–156.

[29] Gazzetta Ufficiale Della Repubblica Italiana. Piano Nazionale Prevenzione Vaccinale (PNPV) 2017-2019; 2017 [accessed 2020 Feb 19]. http://www.trovanorme.salute.gov.it/norme/renderPdf.spring?seriegu=SG&datagu=18/02/2017&redaz=17A01195&artp=1&art=1&subart=1&subart1=10&vers=1&prog=001.

[30] European Centre for Disease Prevention and Control (ECDC). Survey of healthcare workers’ knowledge, attitudes and behaviours on antibiotics, antibiotic use and antibiotic resistance in the EU/EEA; 2019 [accessed 2020 Feb 23]. https://www.ecdc.europa.eu/sites/default/files/documents/survey-of-healthcare-workers-knowledge-attitudes-behaviours-on-antibiotics.pdf.

[31] Krockow EM, Colman AM, Chattoe-Brown E, Jenkins DR, Perera N, Mehtar S, Tarrant C. Balancing the risks to individual and society: a systematic review and synthesis of qualitative research on antibiotic prescribing behaviour in hospitals. J Hosp Infect. 2019;101(4):428–39. doi:10.1016/j.jhin.2018.08.007. 30099092

[32] Salsgiver E, Bernstein D, Simon MS, Eiras DP, Greendyke W, Kubin CJ, Mehta M, Nelson B, Loo A, Ramos LG, et al. Knowledge, attitudes, and practices regarding antimicrobial use and stewardship among prescribers at acute-care hospitals. Infect Control Hosp Epidemiol. 2018;39(3):316–22. doi:10.1017/ice.2017.317. 29402339

[33] Labricciosa FM, Sartelli M, Correia S, Abbo LM, Severo M, Ansaloni L, Coccolini F, Alves C, Melo RB, Baiocchi GL, et al. Emergency surgeons’ perceptions and attitudes towards antibiotic prescribing and resistance: a worldwide cross-sectional survey. World J Emerg Surg. 2018;13(1):27. doi:10.1186/s13017-018-0190-5. 29988647 PMC6027784

[34] Malarski M, Hasso-Agopsowicz M, Soble A, Mok W, Mathewson S, Vekemans J. Vaccine impact on antimicrobial resistance to inform Gavi, the vaccine alliance’s 2018 vaccine investment strategy: report from an expert survey. F1000Res. 2019;8:1685. doi:10.12688/f1000research.20100.1. 31737260 PMC6807152

[35] van de Beek D, Cabellos C, Dzupova O, Esposito S, Klein M, Kloek AT, Leib SL, Mourvillier B, Ostergaard C, Pagliano P, et al. ESCMID guideline: diagnosis and treatment of acute bacterial meningitis. Clin Microbiol Infect. 2016;22(Suppl 3):S37–62. doi:10.1016/j.cmi.2016.01.007. 27062097

[36] Igidbashian S, Bertizzolo L, Tognetto A, Azzari C, Bonanni P, Castiglia P, Conversano M, Esposito S, Gabutti G, Icardi G, et al. Invasive meningococcal disease in Italy: from analysis of national data to an evidence-based vaccination strategy. J Prev Med Hyg. 2020;61(2). E152–E161. 32802999 10.15167/2421-4248/jpmh2020.61.2.1589 PMC7419122

[37] Surveillance on antimicrobial resistance, 2018. European Center for Disease Control and Prevention (ECDC). [accessed 2021 Apr 14]. https://www.ecdc.europa.eu/sites/default/files/documents/surveillance-antimicrobial-resistance-Europe-2018.pdf.

[38] Siani A. Measles outbreaks in Italy: a paradigm of the re-emergence of vaccine-preventable diseases in developed countries. Prev Med. 2019;121:99–104. doi:10.1016/j.ypmed.2019.02.011. 30763627

[39] Morbillo e Rosolia News, Rapporto n.37, Gennaio 2018. Istituto Superiore di Sanità. [accessed 2021 Apr 14]. https://www.epicentro.iss.it/morbillo/bollettino/RM_News_2018_37%20def.pdf.

[40] Bechini A, Boccalini S, Baldo V, Cocchio S, Castiglia P, Gallo T, Giuffrida S, Locuratolo F, Tafuri S, Martinelli D, et al. Impact of universal vaccination against varicella in Italy. Hum Vaccin Immunother. 2015;11(1):63–71. doi:10.4161/hv.34311. 25483517 PMC4514224

[41] Bernal JL, Hobbelen P, Amirthalingam G. Burden of varicella complications in secondary care, England, 2004 to 2017. Euro Surveill. 2019;24:42. doi:10.2807/1560-7917.ES.2019.24.42.1900233. PMC6807256 31640840

[42] Kandeil W, Atanasov P, Avramioti D, Fu J, Demarteau N, Li X. The burden of pertussis in older adults: what is the role of vaccination? a systematic literature review. Expert Rev Vaccines. 2019;18(5):439–55. doi:10.1080/14760584.2019.1588727. 30887849

[43] Blasi F, Bonanni P, Braido F, Gabutti G, Marchetti F, Centanni S. The unmet need for pertussis prevention in patients with chronic obstructive pulmonary disease in the Italian context. Hum Vaccin Immunother. 2020;16(2):340–48. doi:10.1080/21645515.2019.1652517. 31403385 PMC7062424

[44] European Centre of Disease Control and Prevention (ECDC). Surveillance atlas of infectious diseases - Pertussis Italy 2018. [accessed 2021 Apr 15]. https://atlas.ecdc.europa.eu/public/index.aspx.

[45] European Centre for Disease Prevention and Control (ECDC). Pertussis annual epidemiological report for 2018. [accessed 2021 May 10]. https://www.ecdc.europa.eu/en/publications-data/pertussis-annual-epidemiological-report-2018.

[46] Statistiche Demografiche - Italia. Popolazione per età, sesso e stato civile 2019. [accessed 2021 May 10]. https://www.tuttitalia.it/statistiche/popolazione-eta-sesso-stato-civile-2019/.

[47] Chen CC, Balderston McGuiness C, Krishnarajah G, Blanchette CM, Wang Y, Sun K, Buck PO. Estimated incidence of pertussis in people aged <50 years in the United States. Hum Vaccin Immunother. 2016;12(10):2536–45. 27246119 10.1080/21645515.2016.1186313 PMC5085009

[48] L’uso degli antibiotici in Italia. Rapporto nazionale 2018. Agenzia Italiana del Farmaco. [accessed 2021 Apr 14]. http://www.salute.gov.it/imgs/C_17_pubblicazioni_2894_allegato.pdf.

[49] Hasso-Agopsowicz M, Prudden H, Vekemans J, World Health Organization. Value attribution framework for vaccines against antimicrobial resistance; 2019 [accessed 2021 Feb 26]. https://www.who.int/immunization/research/meetings_workshops/5_Hasso_Prudden_AMR_PDVAC_2019.pdf?ua=1.

[50] Rossato L, Negrao FJ, Simionatto S. Could the COVID-19 pandemic aggravate antimicrobial resistance? Am J Infect Control. 2020;48(9):1129–30. doi:10.1016/j.ajic.2020.06.192. 32603851 PMC7320258

[51] Rawson TM, Ming D, Ahmad R, Moore LSP, Holmes AH. Antimicrobial use, drug-resistant infections and COVID-19. Nat Rev Microbiol. 2020;18(8):409–10. doi:10.1038/s41579-020-0395-y. 32488173 PMC7264971

[52] Lucien MAB, Canarie MF, Kilgore PE, Jean-Denis G, Fénélon N, Pierre M, Cerpa M, Joseph GA, Maki G, Zervos MJ, et al. Antibiotics and antimicrobial resistance in the COVID-19 era: perspective from resource-limited settings. Int J Infect Dis. 2021;104:250–54. doi:10.1016/j.ijid.2020.12.087. 33434666 PMC7796801

[53] Rawson TM, Wilson RC, Holmes A. Understanding the role of bacterial and fungal infection in COVID-19. Clin Microbiol Infect. 2021;27(1):9–11. doi:10.1016/j.cmi.2020.09.025. 32979569 PMC7546203

[54] Pelfrene E, Botgros R, Cavaleri M. Antimicrobial multidrug resistance in the era of COVID-19: a forgotten plight? Antimicrob Resist Infect Control. 2021;10(1):21. doi:10.1186/s13756-021-00893-z. 33514424 PMC7844805

[55] Zhu N, Aylin P, Rawson T, Gilchrist M, Majeed A, Holmes A. Investigating the impact of COVID-19 on primary care antibiotic prescribing in North West London across two epidemic waves. Clin Microbiol Infect. 2021;27(5):762–68. doi:10.1016/j.cmi.2021.02.007. PMC7884225 33601010

[56] Monnet DL, Harbarth S. Will coronavirus disease (COVID-19) have an impact on antimicrobial resistance? Euro Surveill. 2020;25:45. doi:10.2807/1560-7917.ES.2020.25.45.2001886. PMC7667630 33183403

