# Supplementary material for: Survey among Italian experts on existing vaccines’ role in limiting antibiotic resistance
Source: Hum Vaccin Immunother. 2021 Sep 30;17(11):4283–90. doi: 10.1080/21645515.2021.1969853 (PMC8828092; doi:10.1080/21645515.2021.1969853)
Supplement: Supplemental Material [file KHVI_A_1969853_SM6307.docx]

# Supplementary Material

**Manuscript Title: Survey among Italian Experts on Existing Vaccines’ Role in Limiting Antibiotic Resistance**

**Survey among Italian Experts on Existing Vaccines’ Role in Limiting Antibiotic Resistance**

**Author(s):**

Federico Marchetti^a^, Rosa Prato^b^, Pierluigi Viale^c^

**Affiliations:**

1. GSK, Verona, Italy.
2. Department of Medical and Surgical Sciences, University of Foggia; Department of Hygiene, Policlinico Riuniti University Hospital of Foggia, Foggia, Italy
3. IRCCS Policlinico Sant’Orsola, Infectious Disease Unit, Department of Medical and Surgical Sciences, University of Bologna, Italy

**Corresponding author:** Dr. Federico Marchetti, GSK, Via A. Fleming 2, 37135 Verona, Italy; Email: [federico.e.marchetti@gsk.com](mailto:federico.e.marchetti@gsk.com).

## Survey Questionnaire

Dear Doctor,

Antimicrobial resistance (AMR) is considered a health emergency also in our country. According to the World Health Organization, the vaccines available today are a valid tool to fight AMR because they can help reduce the consumption of antibiotics. However, only influenza and pneumococcal vaccinations are accompanied by clinical trials and meta-analyses that confirm the reduction of antibiotic use in vaccinated communities.

We therefore ask you to give us 10 minutes of your time, to answer a few questions about the value you attribute to four vaccinations (currently available) as a tool to combat AMR, with particular reference to bacterial resistance to antibiotics (ABR).

1. **Could you please indicate your age in years? _______**
2. **Could you indicate the geographic area where you work?**

- Northwest (Piemonte, Valle d’Aosta, Liguria, Lombardia)
- Northeast (Emilia-Romagna, Veneto, Trentino, Friuli-Venezia-Giulia)
- Center (Toscana, Lazio, Umbria, Marche)
- South and islands (Sardegna, Sicilia, Campania, Calabria, Puglia, Basilicata, Abruzzo, Molise)

1. **Could you specify your professional field?**

- Hygiene and Preventive Medicine
- Hospital Pediatrics
- Family Pediatrics
- Infectious Diseases
- Microbiology
- Other

1. **On a personal level, how much of a priority do you give to ABR?**

- ABR is an issue of primary importance in my daily professional activity.
- ABR is an issue that I will soon have to consider.
- ABR is a low priority issue.
- ABR is not in my priority issue

1. **How often do you discuss about the vaccines included in the PNPV (and the National Vaccination Calendar within PNPV) with colleagues, patients, and families?**

- Very often
- Often
- Sometimes
- Infrequently

**We will now ask you to express your opinion on the role of certain vaccines against ABR:**

1. **Pertussis vaccination can limit ABR if:** (check only one box)

- all individuals (healthy or with clinical conditions at risk) included in the National Vaccination Calendar are vaccinated.
- only patients who have been/will be hospitalized (for any clinical cause) and who are therefore at higher risk of nosocomial infection are vaccinated.
- I do not attribute to this vaccine any role against ABR.
- I do not know.

1. **Express with a number from 1 (minimum) to 10 (maximum) the role against ABR that you attribute to pertussis vaccination:**
2. **Meningococcal vaccination (MenB and MenACWY) can limit ABR if:** (check only one box)

- all individuals (healthy or with clinical conditions at risk) included in the National Vaccination Calendar are vaccinated.
- only patients who have been/will be hospitalized (for any clinical cause) and who are therefore at higher risk of nosocomial infection are vaccinated.
- I do not attribute to this vaccine any role against ABR.
- I do not know.

1. **Express with a number from 1 (minimum) to 10 (maximum) the role against ABR that you attribute to meningococcal (MenB and MenACWY) vaccination:**
2. **Measles vaccination can limit ABR if:** (check only one box)

- all individuals (healthy or with clinical conditions at risk) included in the National Vaccination Calendar are vaccinated.
- only patients who have been/will be hospitalized (for any clinical cause) and who are therefore at higher risk of nosocomial infection are vaccinated.
- I do not attribute to this vaccine any role against ABR.
- I do not know.

1. **Express with a number from 1 (minimum) to 10 (maximum) the role against ABR that you attribute to measles vaccination:**
2. **Varicella vaccination can limit ABR if:** (check only one box)

- all individuals (healthy or with clinical conditions at risk) included in the National Vaccination Calendar are vaccinated.
- only patients who have been/will be hospitalized (for any clinical cause) and who are therefore at higher risk of nosocomial infection are vaccinated.
- I do not attribute to this vaccine any role against ABR.
- I do not know.

1. **Express with a number from 1 (minimum) to 10 (maximum) the role against ABR that you attribute to varicella vaccination:**
2. **Overall, do you believe that the vaccines included in the National Vaccination Calendar act against ABR?**

- Yes, all calendar vaccines.
- Yes, but only those with evidence-based documentation (influenza and pneumococcal).
- Yes, both the evidence-based ones and those included in this survey (pertussis, meningococcal, measles, varicella).
- No, only future vaccines against multi-resistant bacteria will be able to fight ABR.
- No vaccination can counteract even partially ABR.
- Not sure.

1. **Do you believe that the role against ABR of the vaccines included in the National Vaccination Calendar should be made explicit in the PNPV and in the PNCAR?**

- Yes, in both Plans
- Yes, but only in PNPV
- Yes, but only in the PNCAR
- No, in neither of both
- I don't know

1. **Do you think that the Scientific Societies should take an official position on the role of the National Vaccination Calendar against ABR?**

- Yes
- No
- I don't know


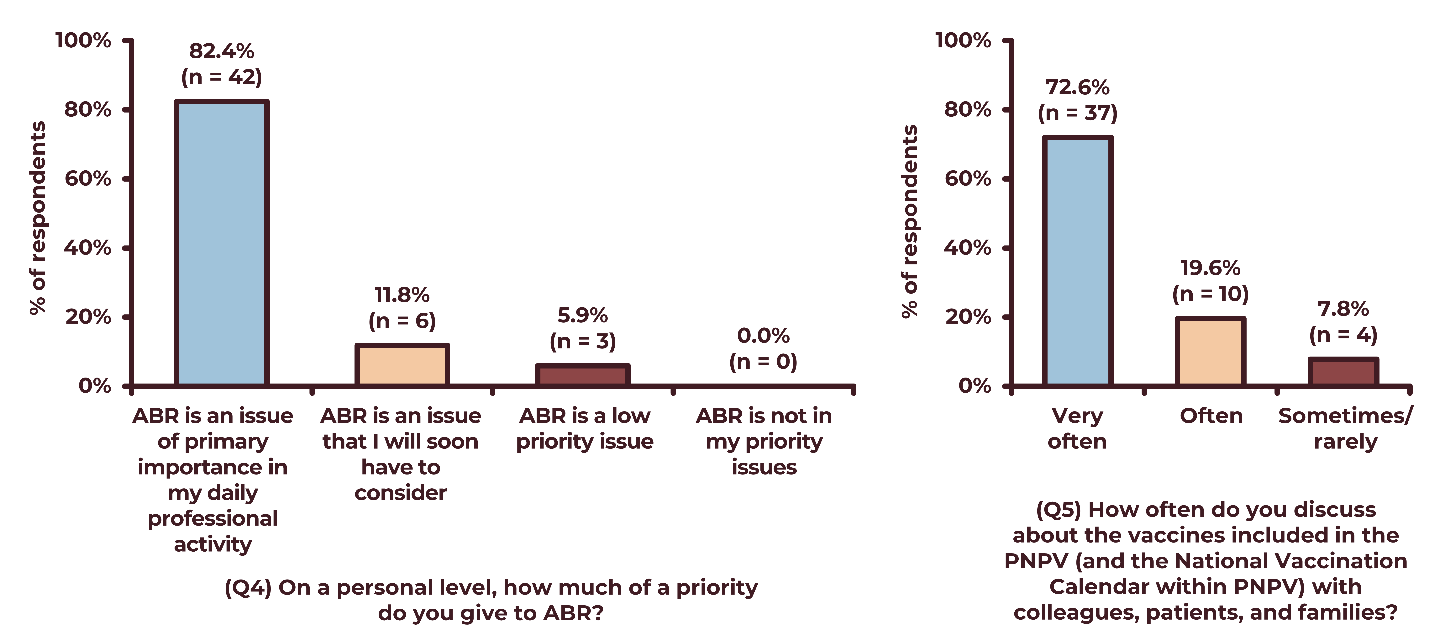


B

A

**Supplementary Figure 1.** ABR and vaccines’ involvement in respondents’ daily professional activity. A) ABR priority level in respondents’ daily professional activity. B) Vaccines’ contribution in respondents’ professional activity. ABR antibiotic resistance.

A


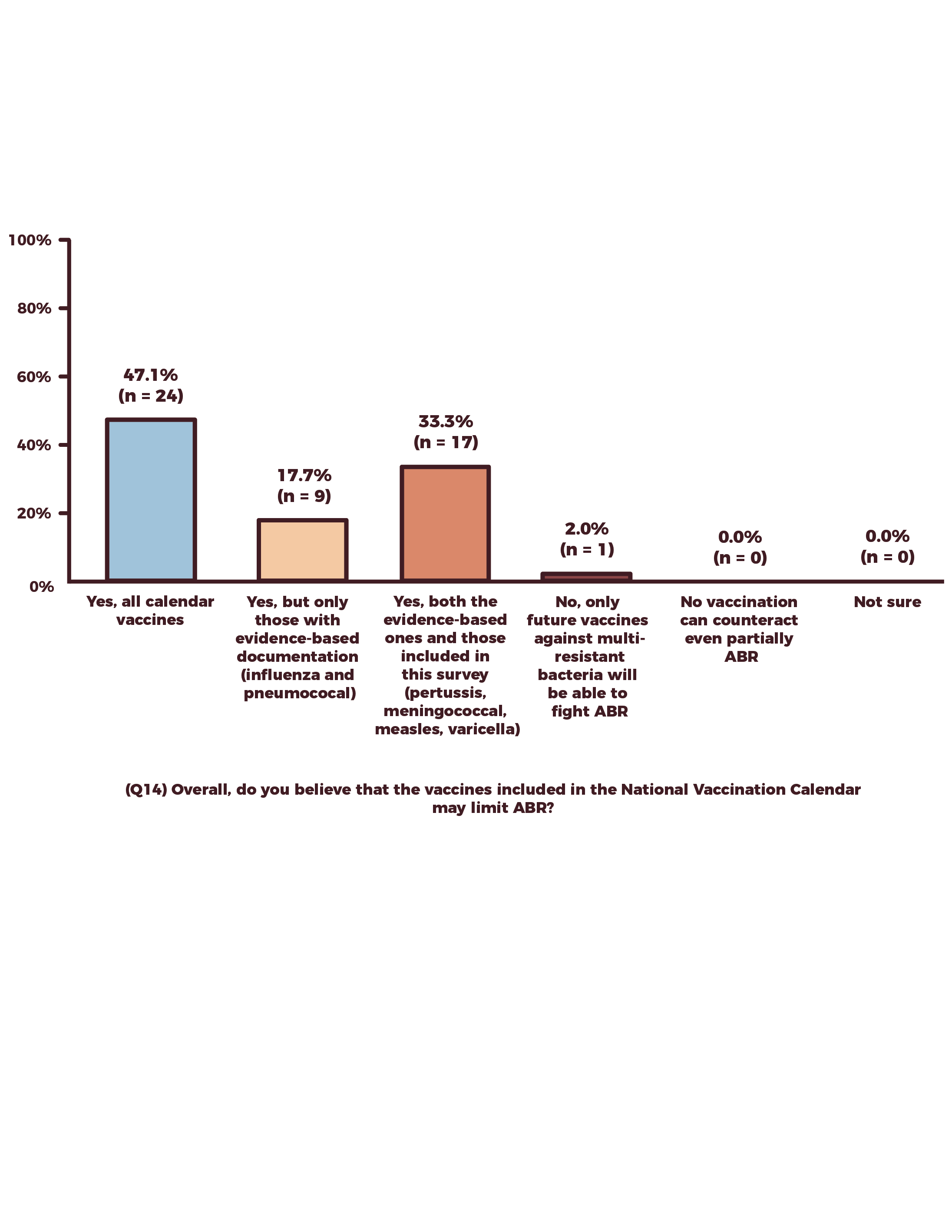


B


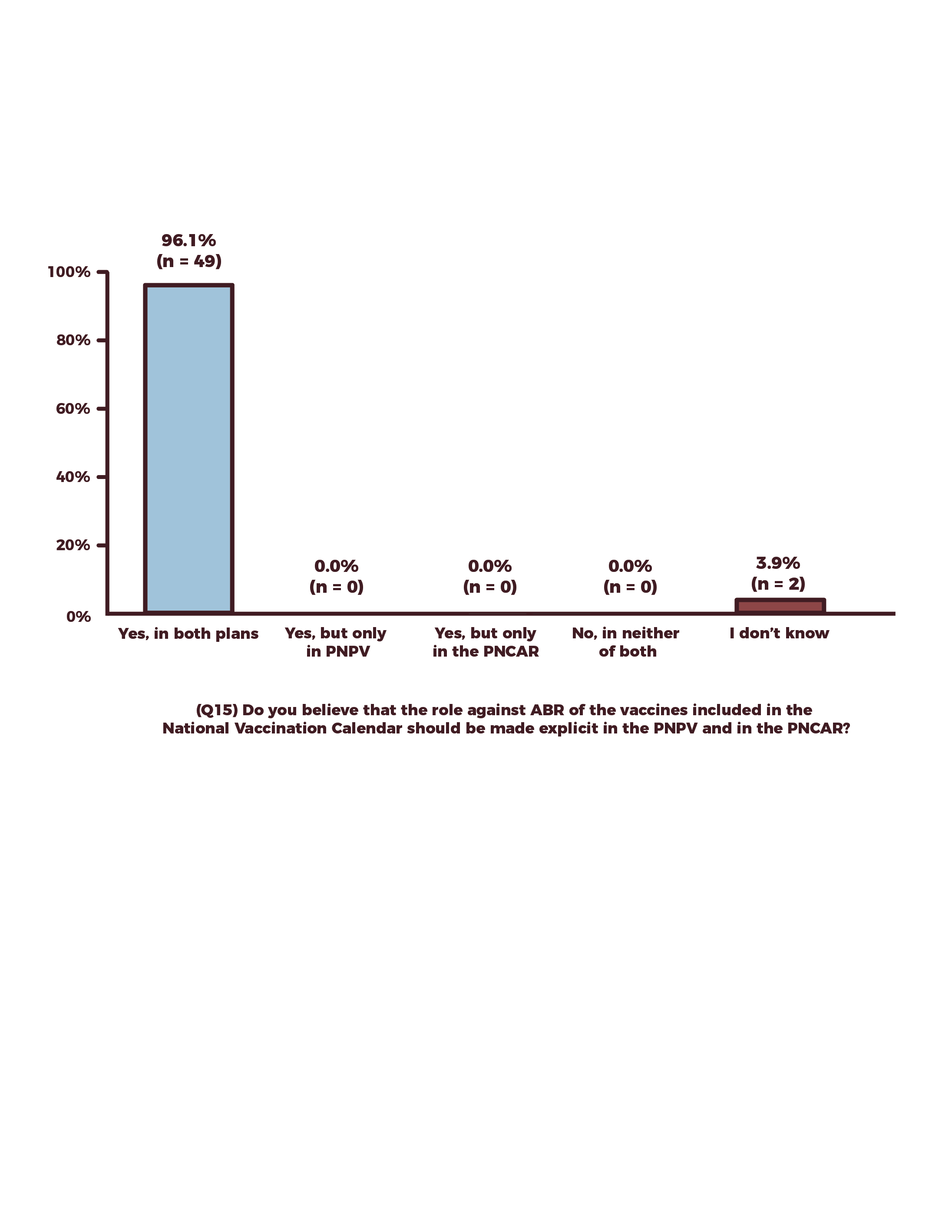


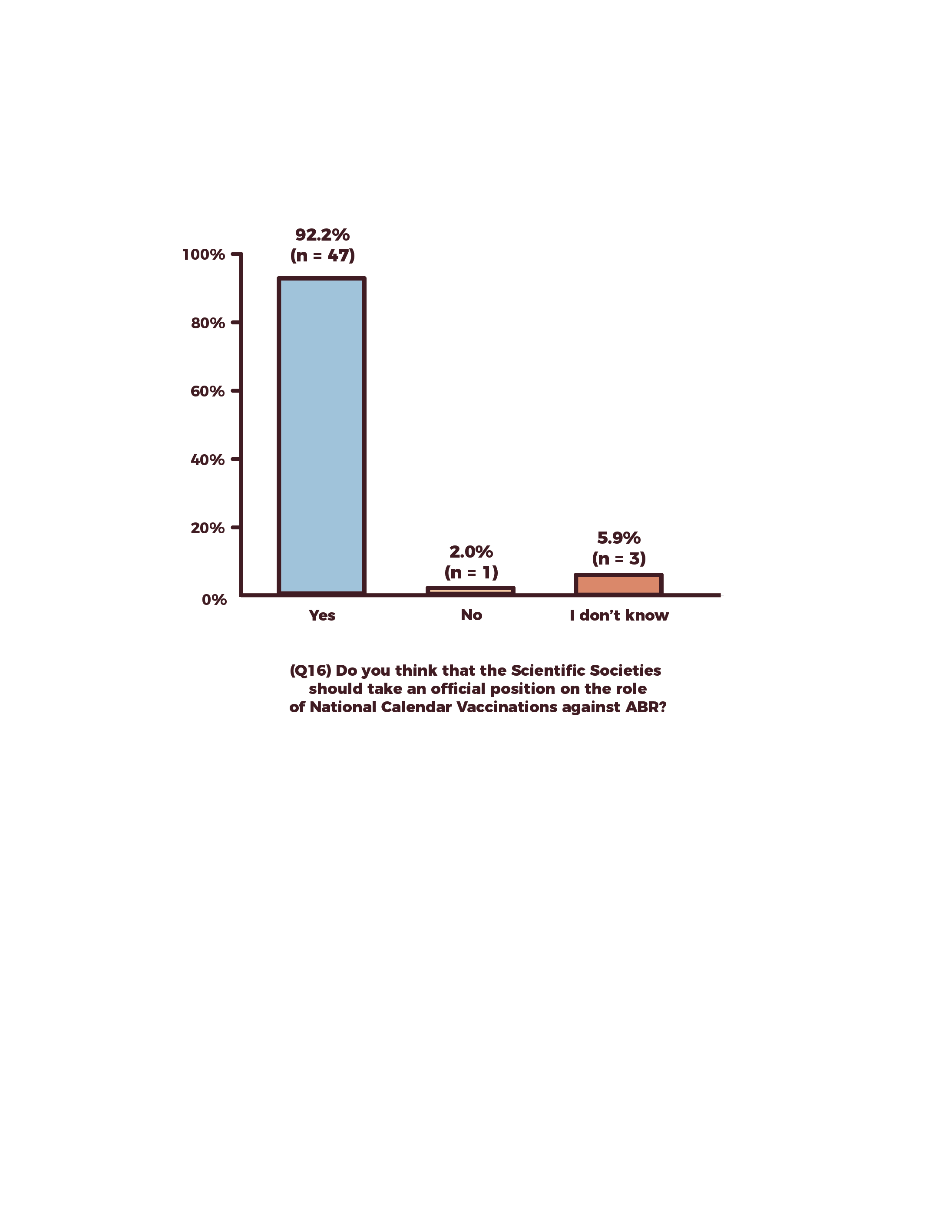
C

**Supplementary Figure 2.** PNPV and ABR. A) Overall role of (all) vaccines against ABR. B) PNPV and PNCAR. C) Scientific Societies on the role of the National Vaccination Calendar against ABR. ABR, antibiotic resistance; PNCAR, Piano Nazionale di Contrasto dell’Antimicrobico-Resistenza [National Action Plan to Combat Antimicrobial Resistance]; PNPV, Piano Nazionale Prevenzione Vaccinale [National Preventive Vaccination Plan].
